# Predictive value of pre-procedural QTc interval for in-hospital major adverse cardiovascular events following primary percutaneous coronary intervention in acute myocardial infarction

**DOI:** 10.3389/fmed.2026.1952189

**Published:** 2026-09-18

**Authors:** Chunyan Wei, Hong'er Li

**Affiliations:** Department of Function, Affiliated Xiaoshan Hospital, Hangzhou Normal University, Hangzhou, China

**Keywords:** acute myocardial infarction, left ventricular ejection fraction, major adverse cardiovascular events, percutaneous coronary intervention, QTc interval, risk stratification

## Abstract

**Background:**

The relationship between pre-procedural corrected QT (QTc) interval and in-hospital major adverse cardiovascular events (MACE) following primary percutaneous coronary intervention (PCI) for acute myocardial infarction (AMI) remains incompletely understood. This study evaluated the predictive value of pre-procedural QTc for in-hospital MACE.

**Methods:**

This single-center retrospective cohort study consecutively enrolled AMI patients undergoing primary PCI. QTc intervals were manually measured from pre-procedural 12-lead electrocardiograms using the Bazett formula, with a prespecified sensitivity analysis using the Fridericia formula. Missing data sensitivity analyses (complete-case BNP model and multiple imputation, m = 20) confirmed that BNP adjustment did not alter the QTc effect. No formal sample size calculation was performed; findings are exploratory. The primary endpoint was in-hospital MACE (composite of ventricular arrhythmias, cardiogenic shock, acute heart failure, and bleeding complications). Logistic regression and receiver operating characteristic (ROC) analyses were performed.

Among 123 patients (mean age 55.3 ± 11.9 years; 91.1% male), 53 (43.1%) experienced MACE. Prolonged QTc (>440 ms) was associated with MACE in univariate analysis (OR = 3.01, 95% CI: 1.41–6.39, *P* = 0.004). After multivariate adjustment, reduced LVEF (OR = 0.76 per 5%, *P* = 0.019) and elevated baseline heart rate (OR = 1.57 per 10 bpm, *P* = 0.023) were independent predictors; continuous QTc was not (OR = 1.03 per 10 ms, *P* = 0.605). The model AUC was 0.767 **(**optimism-corrected AUC = 0.782; optimism = −0.014). Adding QTc to conventional risk factors yielded negligible incremental AUC (*Δ* = 0.001, *P* = 0.888). A numerically high MACE rate was observed in the small subgroup with both LVEF <40% and QTc >440 ms (9/11, 81.8%; 95% CI: 48.2%–97.7%), but the LVEF–QTc interaction was not statistically significant (*P* = 0.941), precluding any conclusion of synergistic risk. A prespecified sensitivity analysis excluding isolated bleeding events (cardiovascular MACE = 40/123, 32.5%) confirmed that LVEF remained independently predictive (OR = 0.73, *P* = 0.008) while QTc showed no independent association (*P* = 0.915) and no incremental value (*Δ*AUC = 0.000, *P* = 0.772). A further sensitivity analysis using the Fridericia formula abolished the univariate QTc–MACE association (OR = 1.23, *P* = 0.615 vs. OR = 3.01, *P* = 0.004 with Bazett), indicating that Bazett overcorrection at high heart rates contributed substantially to the apparent univariate signal. BNP adjustment (complete-case and multiple imputation) did not change the QTc coefficient (>1% variation), indicating no residual BNP confounding.

**Conclusions:**

Pre-procedural QTc prolongation is associated with in-hospital MACE but is not an independent predictor after adjustment for conventional risk factors, with limited incremental value (*Δ*AUC = 0.001). A numerically high MACE rate was noted among patients with both reduced LVEF and prolonged QTc, but this observation derives from only 11 patients with a nonsignificant interaction term (*P* = 0.941); it should be regarded as exploratory and requires validation in larger prospective studies.

## 1 Introduction

Acute myocardial infarction (AMI) remains the leading cause of cardiovascular death worldwide. Primary percutaneous coronary intervention (PCI), the gold standard of reperfusion therapy for ST-segment elevation myocardial infarction (STEMI), has significantly reduced acute-phase mortality; however, the incidence of major adverse cardiovascular events (MACE) during hospitalization remains substantial (1–5).

Among the various electrocardiographic parameters, the QTc interval has emerged as a potential risk stratification tool owing to its ease of measurement, low cost, and bedside availability (6). The QTc interval reflects ventricular repolarization duration, and its prolongation indicates increased repolarization heterogeneity. A substantial body of evidence has demonstrated that QTc prolongation is significantly associated with increased risks of all-cause and cardiovascular mortality. In AMI patients, Lin et al. found that a QTc >445 ms in the emergency department independently predicted all-cause death and heart failure readmission within 2 years (HR = 1.03, *P* < 0.001), while Wattoo et al. confirmed a positive correlation between QTc prolongation and both ventricular arrhythmias (rho = 0.658, *P* < 0.001) and in-hospital mortality (rho = 0.314, *P* = 0.04) (7–11).

Several important gaps remain in the existing literature. First, most studies have focused on QTc dispersion rather than a single QTc value, yet QTc dispersion (QTcd) exhibits substantial measurement variability and poor reproducibility. Second, some investigations have been limited by small sample sizes or enrollment of specific subgroups only. Third, evidence remains insufficient regarding whether pre-procedural QTc interval independently predicts in-hospital MACE after accounting for traditional risk factors such as LVEF and diabetes (12–15).

Diabetes mellitus is an important independent risk factor for adverse outcomes in AMI, with a QTc prolongation prevalence ranging from 18% to 44% among diabetic patients. Hyperglycemia may prolong ventricular repolarization through its effects on myocardial ion channel function. However, whether QTc prolongation and diabetes exert a synergistic effect in predicting MACE following PCI has not been established (16–18).

This study aimed to investigate the relationship between pre-procedural QTc interval and in-hospital MACE in patients with AMI undergoing primary PCI, to evaluate its predictive value, and to analyze the interaction between QTc interval, LVEF, and diabetes, with the goal of identifying an adjunctive marker for acute-phase risk stratification.

## 2 Methods

### 2.1 Study design and population

This single-center retrospective cohort study was conducted at Zhejiang Xiaoshan Hospital (Hangzhou, China). Consecutive patients who underwent primary PCI for AMI between July 2023 and February 28, 2026 were enrolled.

Inclusion criteria were: (1) diagnosis of AMI (STEMI or NSTEMI) according to the Fourth Universal Definition of Myocardial Infarction; (2) primary PCI performed, including successful reperfusion with post-procedural TIMI flow grade ≥ 2 and patients who developed the no-reflow phenomenon; and (3) availability of a 12-lead electrocardiogram (ECG) within 24 h before PCI (19–22).

Exclusion criteria were: (1) absence of a pre-procedural ECG suitable for QT interval measurement; (2) pacemaker rhythm; (3) atrial fibrillation or atrial flutter; (4) bundle branch block; (5) history of prior coronary artery bypass grafting; and (6) incomplete clinical data.

This study was approved by the Ethics Committee of Zhejiang Xiaoshan Hospital (Approval No. XSYY-2026-Lun-031). Informed consent was waived owing to the retrospective observational design.

### 2.2 Data collection

Clinical data were extracted from the hospital electronic medical records system by two independent investigators and verified by a third investigator. The following variables were collected: demographic characteristics (age, sex, body mass index), comorbidities [hypertension, diabetes mellitus, hyperlipidemia, current smoking status (never or former vs. current; pack-years were not recorded), prior myocardial infarction, PCI, or stroke], admission vital signs (systolic blood pressure, diastolic blood pressure, heart rate), laboratory parameters (peak troponin I [TnI], B-type natriuretic peptide [BNP], serum creatinine, total cholesterol [TC], triglycerides [TG], low-density lipoprotein cholesterol [LDL-C], high-density lipoprotein cholesterol [HDL-C], glycated hemoglobin [HbA1c], creatine kinase-MB [CK-MB]), procedural characteristics (target vessel, stent parameters, TIMI flow grade), and echocardiographic parameters (LVEF assessed by the modified biplane Simpson method). Coronary angiographic images were exported from the hospital PACS system, and four illustrative cases were purposively selected to span the range of TIMI flow grades, culprit vessels, and MACE outcomes; these cases were not randomly sampled and do not constitute a representative subset; culprit lesions were independently annotated by two interventional cardiologists for figure illustration (23).

### 2.3 Electrocardiographic measurement

Pre-procedural 12-lead ECGs were recorded using a digital 12-lead electrocardiograph (RAGE-12P, Nalong Health Technology, Xiamen, China; sampling rate: 500 Hz; filter settings: 0.05–150 Hz) in accordance with AHA/ACC/HRS recommendations for ECG standardization, with all recordings obtained within 24 h before PCI. The QT interval was manually measured by two experienced cardiologists who were blinded to clinical outcomes. Measurements were performed across all 12 leads, and the maximum QT value was recorded. The end of the T wave was determined using the tangent method, defined as the intersection of the tangent drawn to the steepest downslope of the T wave with the isoelectric TP baseline, in accordance with the AHA/ACC/HRS recommendations (24); for leads exhibiting prominent U waves or biphasic/notched T waves, the T-wave offset was taken at the point where the terminal T wave returned to the TP baseline, and leads in which the offset could not be reliably identified were excluded for that cardiac cycle. In each lead, three consecutive sinus beats were measured and the values were averaged; beats containing premature ventricular contractions or other supraventricular/ventricular ectopy were discarded and replaced by the next eligible sinus beat. The Bazett formula was applied as the primary correction method (QTcB = QT/√RR, where RR in seconds = 60/heart rate). To assess robustness to the choice of correction formula, all primary analyses were repeated using the Fridericia formula (QTcF = QT/RRⁱ⁄3), which is less heart-rate-dependent and has demonstrated superior performance in large population cohorts (25, 26). The two formulae were compared with respect to QTc distribution, reclassification across the 440 ms threshold, and association with MACE. The primary exposure variable was QTc_max. A uniform threshold of 440 ms was used to define QTc prolongation (without sex stratification), a cutoff widely adopted in AMI-related research. Interobserver agreement was assessed using the intraclass correlation coefficient (ICC), which was 0.92 (95% CI: 0.89–0.94), indicating excellent concordance (27, 28).

### 2.4 Endpoint definitions

The primary endpoint was in-hospital MACE, defined as the composite of: (1) ventricular arrhythmias — sustained ventricular tachycardia, ventricular fibrillation, or torsades de pointes requiring electrical cardioversion or defibrillation; (2) cardiogenic shock — systolic blood pressure <90 mmHg persisting for ≥ 30 min or requiring vasoactive agents or mechanical circulatory support; (3) acute heart failure — new-onset or worsening heart failure necessitating intravenous diuretics, inotropic agents, or mechanical ventilation, with Killip class ≥ III; and (4) hemorrhagic complications — including access-site bleeding, gastrointestinal hemorrhage, and other clinically significant bleeding, classified according to BARC type ≥ 2 criteria. All endpoint events were adjudicated by an independent clinical events committee blinded to electrocardiographic measurements.

### 2.5 Statistical analysis

Continuous variables were expressed as mean ± standard deviation or median [interquartile range (IQR)], and categorical variables were presented as frequencies and percentages. Between-group comparisons were performed using the Student's t-test or Mann–Whitney U test for continuous variables and the chi-square test or Fisher's exact test for categorical variables. All six variables in the primary multivariate model (QTc interval, heart rate, age, sex, LVEF, and diabetes) had zero missing values; thus, the effective sample size for the primary analysis was 123 (100%), and the EPV remained 8.8. Some secondary laboratory parameters (e.g., troponin I, BNP, creatinine, HbA1c, and lipids) had missingness ranging from 26.8% to 80.5%; because these variables were not included in the primary model, listwise deletion did not reduce the multivariate sample size. To assess potential residual confounding by BNP—the only laboratory variable significant in univariate analysis—we performed (i) a complete-case seven-variable model including BNP and (ii) multiple imputation by chained equations (m = 20), as described below.

Both continuous QTc (per 10 ms increment) and dichotomous QTc (>440 ms vs. ≤ 440 ms) were analyzed; the former was entered into the multivariate regression model, and the latter was used for subgroup and combined analyses. Following recommended methodology for clinical prediction model development, candidate predictors were initially screened by univariate logistic regression. Variables with *P* < 0.10 or established clinical relevance (age and sex were forced into the model *a priori* as conventional confounders regardless of statistical significance) were entered into a multivariate model using forward stepwise selection with entry threshold *P* < 0.10 and removal threshold *P* ≥ 0.05. Smoking met the univariate screening criterion (*P* = 0.070) but was not retained because it was strongly collinear with age and sex and its effect was attenuated after adjustment; BNP was significantly associated with MACE in univariate analysis (OR = 1.90, *P* = 0.021) but was excluded from the primary model because 43.1% of values were missing (addressed by separate complete-case and multiple-imputation sensitivity analyses). We also considered least absolute shrinkage and selection operator (LASSO) regression, which may outperform stepwise selection when EPV is low; however, given that (i) only six zero-missingness variables were available for the primary model and (ii) LASSO coefficient estimates are inherently shrinkage-biased and less readily interpretable as adjusted ORs, forward stepwise was selected as the primary approach. The EPV limitation (8.8 < 10) of the stepwise model is explicitly acknowledged in the following sentence. Multicollinearity was assessed using the variance inflation factor (VIF) (all VIF < 5, indicating no severe multicollinearity). Results were expressed as odds ratios (OR) with 95% confidence intervals (CI). The multivariate model included 6 independent variables with 53 MACE events, yielding an events-per-variable (EPV) ratio of 8.8, which falls below the conventionally recommended EPV ≥ 10 and the EPV ≥ 20 threshold proposed by Riley et al., indicating that results should be interpreted with caution. No formal *a priori* sample size or power calculation was performed; this retrospective study enrolled all consecutive eligible patients during the study period, and the analyses should be considered exploratory (29, 30).

Discriminative ability was assessed by the area under the receiver operating characteristic curve (AUC), and calibration was evaluated using the Hosmer-Lemeshow test. The Youden index was used to determine the optimal cutoff value. Patients were stratified into quartiles based on QTc, and the linear trend in MACE incidence was analyzed using the Cochran-Armitage trend test. Subgroup analyses were conducted according to key clinical characteristics, with interaction terms included to assess effect modification. To address the potential heterogeneity introduced by including bleeding complications in the composite endpoint, a prespecified sensitivity analysis was performed using a “cardiovascular MACE” endpoint that excluded patients whose sole adverse event was hemorrhagic (BARC ≥ 2), retaining only ventricular arrhythmias, cardiogenic shock, and acute heart failure; patients with concurrent cardiovascular events and bleeding were retained in the cardiovascular MACE group. All primary analyses (univariate and multivariate logistic regression, ROC analysis, quartile analysis, subgroup analysis, and combined LVEF-QTc analysis) were repeated using this redefined endpoint. This study adhered to the STROBE statement. All statistical analyses were performed using Python 3.10, and a two-sided *P* < 0.05 was considered statistically significant. A second prespecified sensitivity analysis was conducted using the Fridericia-corrected QTc (QTcF) in place of the Bazett-corrected QTc (QTcB); the same 440 ms threshold and all primary analyses were applied to QTcF for both the full MACE and cardiovascular MACE endpoints (31, 32).

## 3 Results

### 3.1 Study population and baseline characteristics

A total of 142 patients who underwent primary PCI for AMI were initially screened. After excluding 19 patients without pre-procedural ECG records suitable for QT interval measurement, 123 patients were included in the final analysis (Figure 1).

**Figure 1:**
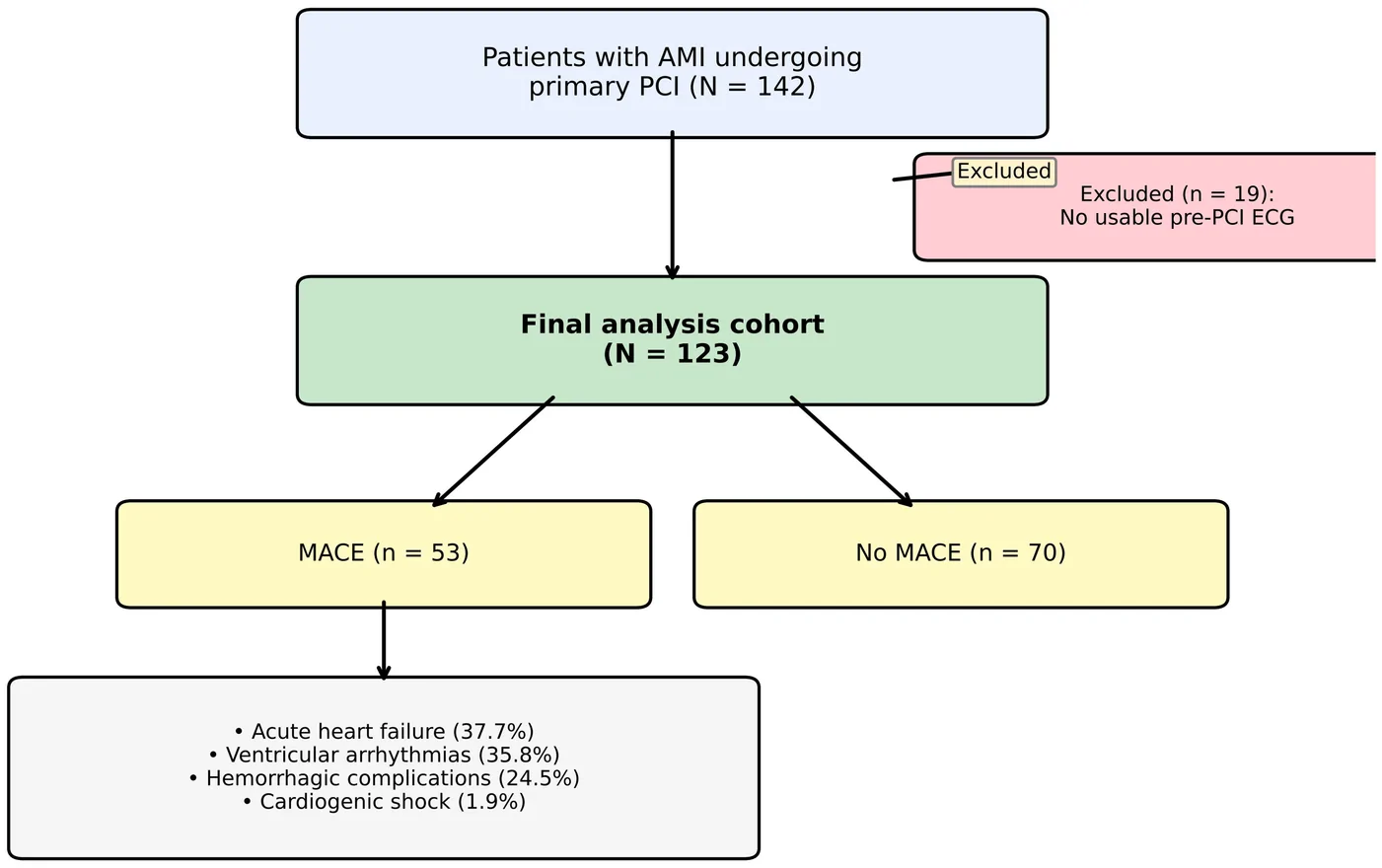
Patient selection flowchart. A total of 142 patients who underwent primary percutaneous coronary intervention (PCI) for acute myocardial infarction were initially enrolled in this study. After excluding 19 patients without pre-PCI electrocardiographic records suitable for QT interval measurement, 123 patients were included in the final analysis. MACE, major adverse cardiovascular events.

Baseline clinical characteristics are summarized in Table 1. The mean age of the study population was 55.3 ± 11.9 years, with 112 males (91.1%). The prevalence of hypertension, diabetes mellitus, and hyperlipidemia was 70.7%, 30.9%, and 42.3%, respectively. The median LVEF was 51.0% (IQR: 43.0–58.0%), and the median pre-procedural QTc interval was 437.0 ms (IQR: 417.5–460.0 ms).

**Table 1:** Baseline characteristics of the study population.

Variable	Total (*N* = 123)	No MACE (*n* = 70)	MACE (*n* = 53)	*P* value
Age, years	55.3 ± 11.9	54.9 ± 11.7	55.8 ± 12.4	0.685
Male sex, yes, n (%)	112 (91.1)	63 (90.0)	49 (92.5)	0.878
SBP, mmHg	130.3 ± 19.5	128.9 ± 20.2	132.2 ± 18.6	0.346
DBP, mmHg	82.0 (75.0–92.8)	82.0 (74.0–90.0)	83.0 (76.0–96.0)	0.271
Heart rate (discharge)†, bpm	84.1 ± 13.3	82.3 ± 12.3	86.3 ± 14.3	0.108
Troponin I, ng/mL	14.3 (1.5–73.2)	8.0 (1.0–60.2)	35.5 (3.6–82.0)	0.086
Creatinine, µmol/L	82.5 (71.2–117.1)	79.2 (71.5–91.8)	91.4 (71.7–123.4)	0.296
LDL-C, mmol/L	3.2 (2.6–3.7)	3.2 (2.9–3.7)	3.2 (2.0–3.7)	0.574
HDL-C, mmol/L	0.9 (0.8–1.0)	0.9 (0.8–0.9)	0.9 (0.8–1.0)	0.664
BNP, pg/mL	246.0 (136.4–429.0)	179.4 (127.2–274.9)	275.0 (150.9–475.1)	0.046
LVEF, %	51.0 (43.0–58.0)	54.0 (46.0–59.0)	47.0 (39.0–54.8)	<0.001
QTc max, ms	437.0 (417.5–460.0)	432.5 (409.0–450.0)	447.0 (422.0–464.0)	0.027
Pre-procedural HR‡, bpm	70.0 (64.0–81.0)	68.0 (63.0–74.8)	75.0 (67.0–87.0)	<0.001
Pre-procedural QRS, ms	96.0 (92.0–101.5)	96.0 (91.0–101.8)	96.0 (92.0–101.0)	0.332
Hypertension, yes, n (%)	87 (70.7)	47 (67.1)	40 (75.5)	0.421
Diabetes, yes, n (%)	38 (30.9)	16 (22.9)	22 (41.5)	0.043
Hyperlipidemia, yes, n (%)	52 (42.3)	29 (41.4)	23 (43.4)	0.973
Smoking, yes, n (%)	12 (9.8)	10 (14.3)	2 (3.8)	0.101
QTc >440 ms, yes, n (%)	47 (38.2)	19 (27.1)	28 (52.8)	0.007

Data are presented as mea*n* ± SD or median (IQR) for continuous variables, and n (%) for categorical variables. *P* values from independent t-test or Mann–Whitney U test for continuous variables, chi-square test for categorical variables. *P < 0.05. †Discharge heart rate, reflecting vital sign status at discharge; not used in predictive model analyses. ‡ Pre-procedural heart rate (before PCI), the core analytical variable in this study, used in univariate and multivariate logistic regression analyses. The two heart rate variables reflect clinical status at different time points and should be distinguished accordingly.

During hospitalization, 53 patients (43.1%) experienced MACE. Classified by primary event type (each patient counted under the most clinically significant category only), acute heart failure occurred in 20 patients (37.7% of those with MACE), ventricular arrhythmias in 19 (35.8%; including ventricular fibrillation in 14 episodes and ventricular tachycardia in 7 episodes, with some patients experiencing multiple event types), hemorrhagic complications in 13 (24.5%), and cardiogenic shock in 1 (1.9%). (Figure 2A) Additional patients experienced multiple concurrent MACE events (e.g., heart failure complicated by bleeding, ventricular fibrillation co-occurring with heart failure) (Figure S2A). Of the 53 MACE patients, 13 (24.5%) had bleeding as their sole adverse event without any cardiovascular endpoint; these patients were excluded in the prespecified sensitivity analysis, yielding a cardiovascular MACE cohort of 40 patients (32.5%).

**Figure 2:**
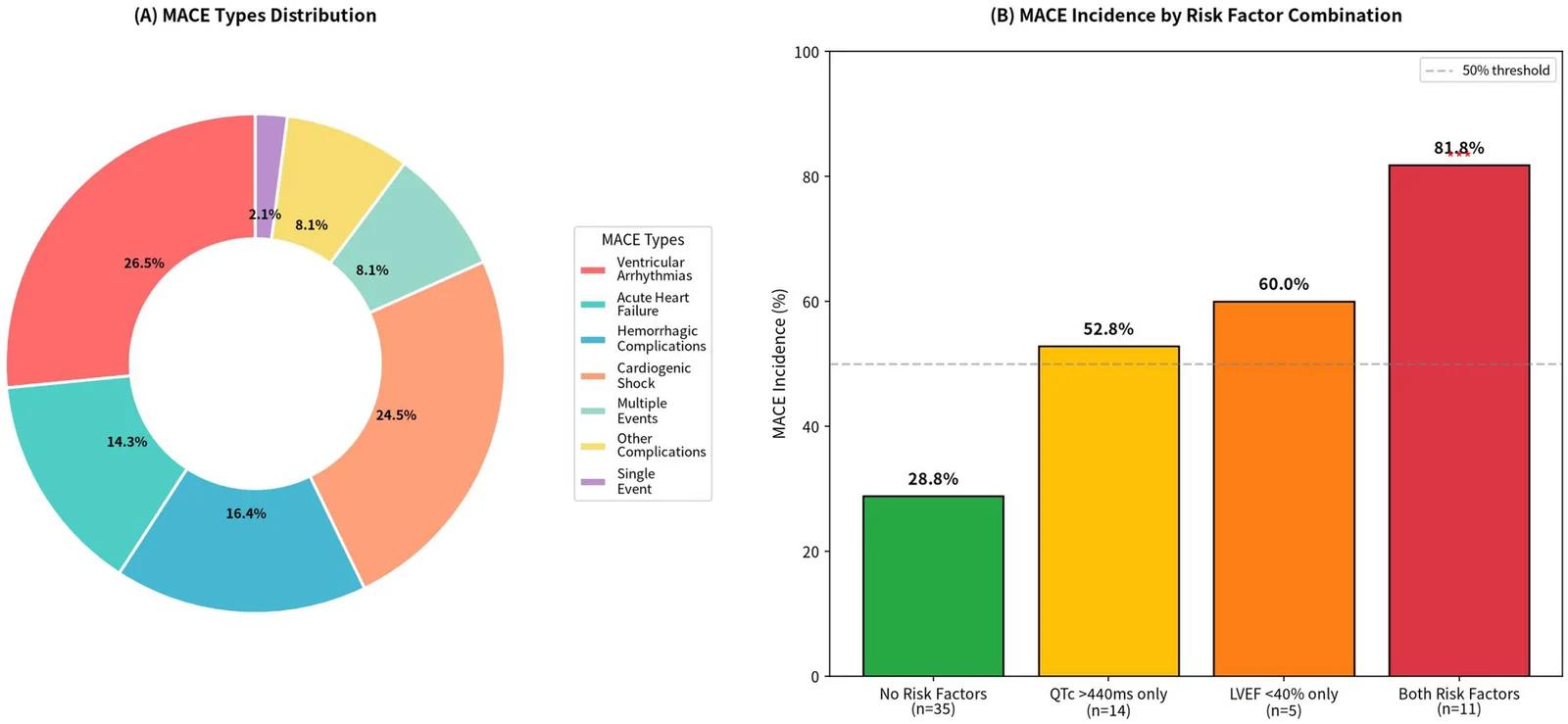
MACE types distribution and interaction between QTc prolongation and diabetes mellitus. **(A)** Pie chart showing the proportional distribution of different MACE types among patients who experienced events (*n* = 53). Acute heart failure accounted for 37.7% of MACE, followed by ventricular arrhythmias (35.8%), hemorrhagic complications (24.5%), and cardiogenic shock (1.9%). **(B)** Bar chart showing MACE incidence stratified by QTc status (>440 ms vs ≤440 ms) and diabetes mellitus status.

### 3.2 Pre-procedural QTc interval distribution

The distribution of pre-procedural QTc intervals is shown in Figure 3. QTc values ranged from 348 to 619 ms, with a median of 437.0 ms (IQR: 417.5–460.0 ms). A total of 47 patients (38.2%) had prolonged QTc (>440 ms), while 76 (61.8%) had normal QTc (≤440 ms).

**Figure 3:**
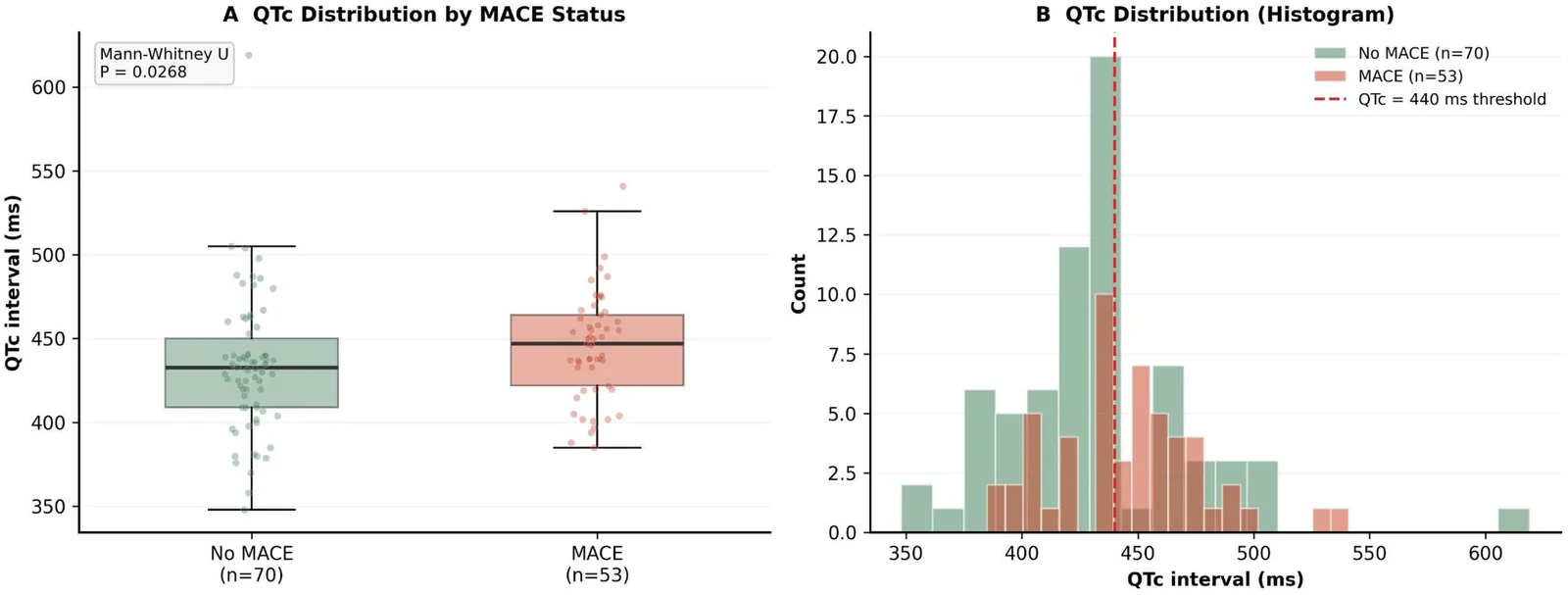
Distribution of pre-PCI QTc intervals. Histogram showing the distribution of corrected QT (QTc) intervals in the study population (*N* = 123). The vertical dashed line indicates the 440 ms cutoff value for QTc prolongation. The density curve is overlaid in blue. In the upper right corner, the number and percentage of patients with QTc >440 ms and ≤440 ms are displayed.

Compared with the normal QTc group, patients with prolonged QTc had a higher proportion of females (17.0% vs. 3.9%, *P* = 0.021), a higher prevalence of diabetes mellitus (46.8% vs. 21.1%, *P* = 0.005), faster heart rates (78.0 ± 14.8 vs. 69.9 ± 9.5 bpm, *P* < 0.001), and lower LVEF (47.6 ± 10.8% vs. 51.4 ± 9.2%, *P* = 0.039). When QTc was recalculated using the Fridericia formula (QTcF = QT/RRⁱ⁄3), the mean QTcF was 426.4 ± 35.4 ms (vs. 438.8 ± 38.0 ms by Bazett; mean difference 12.4 ms), and only 32 patients (26.0%) exceeded 440 ms compared with 47 (38.2%) by Bazett. Notably, 16 patients classified as having prolonged QTc by Bazett were reclassified as normal by Fridericia; these patients had significantly higher heart rates (87.3 ± 9.4 vs. 73.1 ± 14.6 bpm, *P* < 0.001), and the Bazett–Fridericia discrepancy increased with heart rate (mean overestimation: 3.0 ms at HR < 70 bpm, 24.1 ms at HR 80–90 bpm, and 36.0 ms at HR ≥ 90 bpm). QTcB was positively correlated with heart rate (r = 0.287, *P* = 0.001), whereas QTcF showed no significant correlation (r = −0.021, *P* = 0.821), confirming that Bazett overcorrection at elevated heart rates accounted for a substantial portion of the apparent QTc prolongation. No statistically significant differences were observed between the two groups for age, hypertension, or other baseline characteristics (all *P* > 0.05; Figure 4, Table 1).

**Figure 4:**
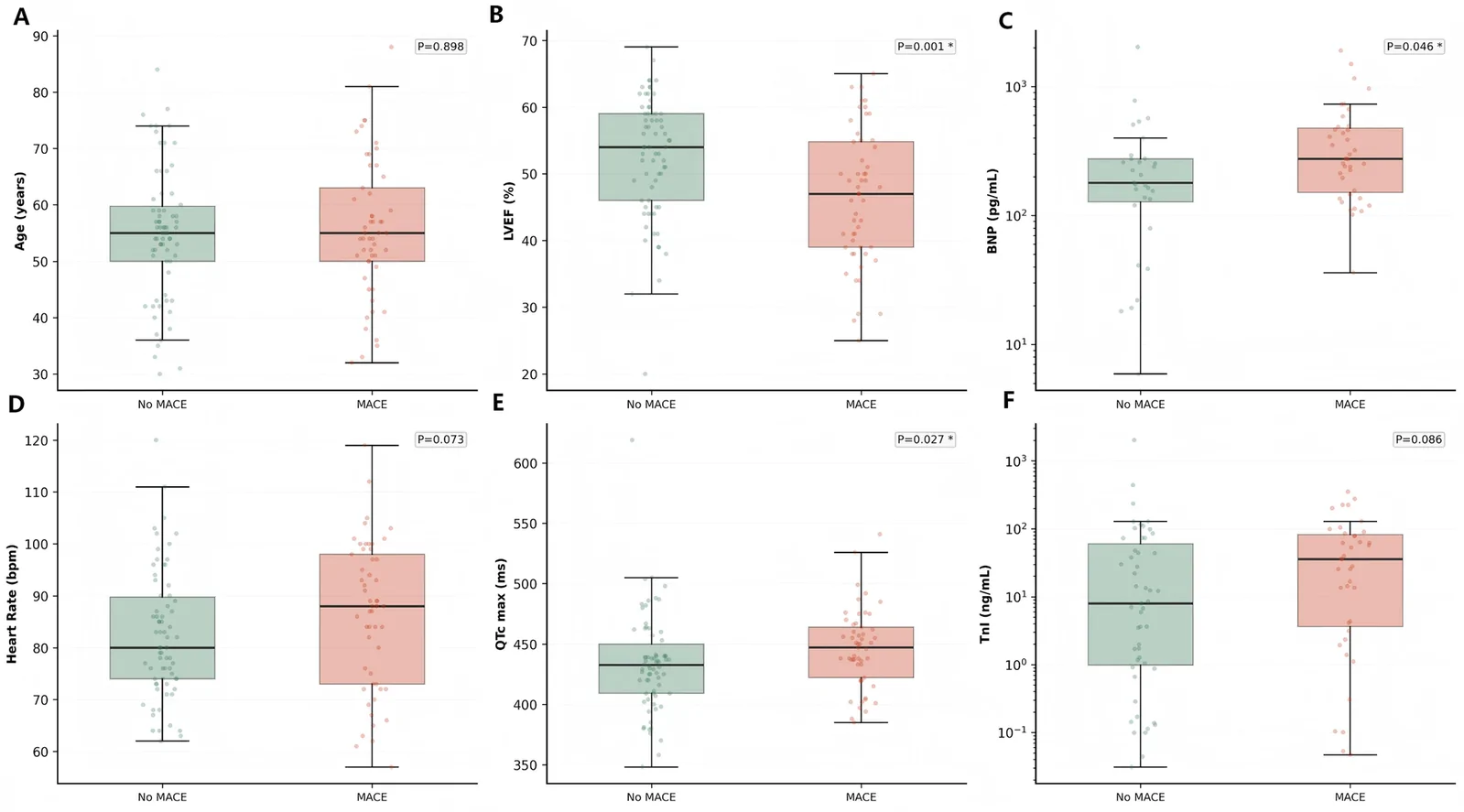
Comparison of baseline characteristics between QTc >440 ms and ≤440 ms groups. Six-panel box plots comparing the distribution of key baseline characteristics between patients with and without QTc prolongation (>440 ms vs. ≤440 ms). Panels show: **(A)** Age, **(B)** Heart rate, **(C)** Left ventricular ejection fraction (LVEF), **(D)** B-type natriuretic peptide (BNP), **(E)** QTc interval, and **(F)** Systolic blood pressure (SBP). *P*-values were calculated using the Mann–Whitney U test or independent t-test as appropriate.

### 3.3 Predictors of MACE: univariate analysis

Univariate logistic regression analysis demonstrated that prolonged QTc (>440 ms) was significantly associated with the risk of MACE (OR = 3.01, 95% CI: 1.41–6.39, *P* = 0.004) (Table 2, Figure 5, left panel). Each 10-ms increment in QTc interval showed a trend toward increased MACE risk, although this did not reach statistical significance (OR = 1.09, 95% CI: 0.99–1.21, *P* = 0.079).

**Table 2:** Univariate and multivariate logistic regression analysis for in-hospital MACE.

Variable	Univariate OR (95% CI)	P	Multivariate OR (95% CI)	P
Age (per 5 yr)	1.03 (0.89–1.20)	0.680	1.03 (0.87–1.24)	0.715
Female sex	0.73 (0.20–2.65)	0.638	0.38 (0.08–1.85)	0.231
Pre-procedural HR (per 10 bpm)	1.80 (1.27–2.54)	0.001	1.57 (1.06–2.31)	0.023
LVEF (per 5%)	0.72 (0.59–0.88)	0.001	0.76 (0.61–0.96)	0.019
BNP (per ln)	1.90 (1.10–3.27)	0.021	-	
Diabetes	2.40 (1.10–5.23)	0.028	2.12 (0.86–5.24)	0.103
Hypertension	1.51 (0.68–3.35)	0.316	-	
Smoking	0.24 (0.05–1.12)	0.070	-	
QTc (per 10 ms)	1.09 (0.99–1.21)	0.079	1.03 (0.92–1.16)	0.605
QTc >440 ms	3.01 (1.41–6.39)	0.004	-	

Multivariate model AUC, 0.767, adjusted for QTc, pre-procedural heart rate, age, sex, LVEF, and diabetes mellitus. OR, odds ratio; CI, confidence interval. * *P* < 0.05. Dashes (—) indicate variables not retained in the final multivariate model: hypertension did not meet the univariate screening threshold (*P* = 0.316); smoking met univariate screening (*P* = 0.070) but was excluded owing to collinearity with age and sex; and BNP was excluded due to a 43.1% missing-data rate, addressed by separate complete-case and multiple-imputation sensitivity analyses.

**Figure 5:**
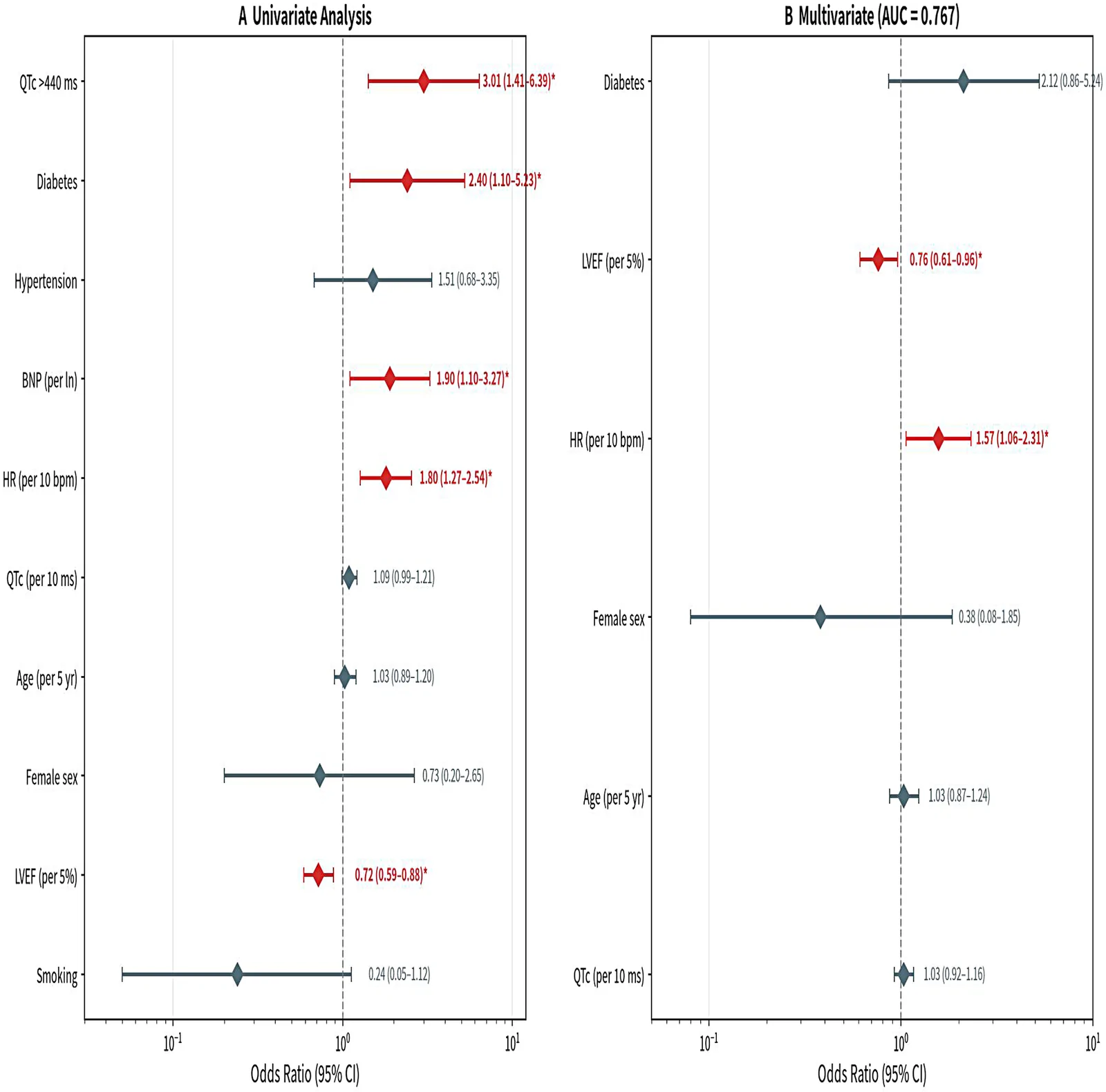
Forest plot of univariate and multivariate logistic regression analysis for in-hospital MACE. Forest plot displaying odds ratios (OR) with 95% confidence intervals (CI) for predictors of in-hospital major adverse cardiovascular events (MACE). The left panel shows results from univariate analysis, and the right panel shows results from multivariate logistic regression adjusted for pre-procedural heart rate, age, sex, left ventricular ejection fraction (LVEF), and diabetes mellitus. The dashed vertical line represents OR = 1. Variables with *P* < 0.05 in multivariate analysis are considered statistically significant. The in-figure label “AUC=0.734” in panel B is a typographical error from an earlier model version; the correct multivariate model AUC is 0.767 as stated in the text and Table 2. The figure has been regenerated accordingly.

Other significant predictors included elevated pre-procedural heart rate (per 10 bpm increment: OR = 1.80, 95% CI: 1.27–2.54, *P* = 0.001), reduced LVEF (per 5% increment: OR = 0.72, 95% CI: 0.59–0.88, *P* = 0.001), elevated BNP (natural log-transformed; n = 70 with available BNP data: OR = 1.90, 95% CI: 1.10–3.27, *P* = 0.021), and diabetes mellitus (OR = 2.40, 95% CI: 1.10–5.23, *P* = 0.028). Age, sex, blood pressure, and other laboratory parameters were not significantly associated with MACE.

### 3.4 Multivariate analysis and predictive model

After adjustment for baseline heart rate, age, sex, LVEF, and diabetes, multivariate logistic regression analysis revealed that reduced LVEF (per 5% increment: OR = 0.76, 95% CI: 0.61–0.96, *P* = 0.019) and elevated baseline heart rate (per 10 bpm increment: OR = 1.57, 95% CI: 1.06–2.31, *P* = 0.023) were independent predictors of in-hospital MACE (Table 2, Figure 5, right panel). Continuous QTc did not achieve independent predictive significance after multivariate adjustment (per 10 ms: OR = 1.03, 95% CI: 0.92–1.16, *P* = 0.605). Diabetes mellitus also did not reach independent significance after multivariate adjustment (OR = 2.12, 95% CI: 0.86–5.24, *P* = 0.103).

The predictive model incorporating QTc interval, baseline heart rate, age, sex, LVEF, and diabetes demonstrated satisfactory discriminative performance, with an AUC of 0.767 (Figure 6). At the optimal cutoff value of 0.453 determined by the Youden index, the model sensitivity was 67.9% and specificity was 81.4%. The Hosmer-Lemeshow test indicated good model calibration (*χ*^2^ = 7.462, *P* = 0.488).

**Figure 6:**
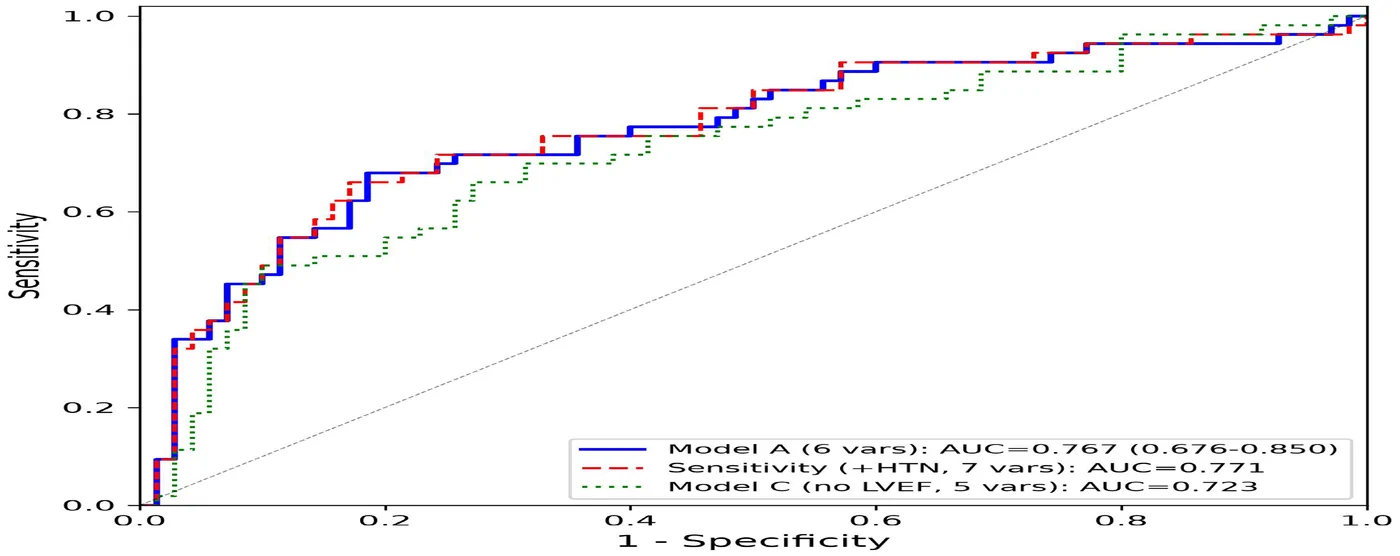
Receiver operating characteristic (ROC) curve for the predictive model. ROC curve illustrating the discriminative ability of the multivariate predictive model incorporating QTc interval, pre-procedural heart rate, age, sex, LVEF, and diabetes mellitus for predicting in-hospital MACE. The area under the curve (AUC) was 0.767. The diagonal reference line represents a non-discriminative model (AUC = 0.5). The optimal cutoff point was determined using the Youden index.

### Sensitivity analysis

To verify the rationale for excluding hypertension, we incorporated hypertension into the multivariate model. The seven-variable model including hypertension yielded an AUC of 0.771 (95% CI: 0.679–0.856), showing no meaningful improvement over Model A (AUC = 0.767). Hypertension was not statistically significant in the model (OR = 1.17, 95% CI: 0.46–2.95, *P* = 0.748), and its inclusion did not improve overall model fit, while reducing the EPV from 8.8 to 7.6. The OR values and significance directions of other variables remained consistent, confirming the robustness of the original model.

To assess the degree of model overfitting, internal validation was performed using bootstrap resampling (2,000 resamples). The mean bootstrap AUC was 0.782 (95% CI: 0.682–0.869), with an optimism value of −0.014 (a negative value indicating no overfitting), and the optimism-corrected AUC was 0.782, suggesting good internal robustness.

Furthermore, to evaluate the incremental predictive value of QTc, a reduced model excluding QTc (incorporating baseline heart rate, age, sex, LVEF, and diabetes only) was constructed and compared with the full model including QTc. The base model AUC was 0.766 (95% CI: 0.669–0.854), and the AUC after adding QTc was 0.767 (95% CI: 0.676–0.850), with a *Δ*AUC of only 0.001 (95% CI: −0.014–0.016, DeLong *P* = 0.888). Category-free NRI was 0.019 (*P* = 0.581), continuous NRI was 0.002 (*P* = 0.649), and IDI was 0.002 (*P* = 0.649), all nonsignificant. These findings indicate that the incremental discriminatory value of QTc beyond conventional risk factors is limited.

#### Sensitivity analysis excluding isolated bleeding events

After excluding 13 patients whose sole endpoint event was hemorrhagic (BARC ≥ 2), the cardiovascular MACE rate was 32.5% (40/123). Prolonged QTc (>440 ms) showed a positive but statistically nonsignificant association with cardiovascular MACE in univariate analysis (OR = 2.07, 95% CI: 0.96–4.49, *P* = 0.064); continuous QTc per 10 ms was not significant (OR = 1.06, 95% CI: 0.96–1.17, *P* = 0.241). In the multivariate model, reduced LVEF remained an independent predictor (OR = 0.73 per 5%, 95% CI: 0.58–0.92, *P* = 0.008), while elevated heart rate showed a borderline association (OR = 1.41 per 10 bpm, 95% CI: 0.96–2.07, *P* = 0.077) and QTc showed no independent association (OR = 0.99 per 10 ms, 95% CI: 0.88–1.12, *P* = 0.915). The full model AUC was 0.768 (95% CI: 0.679–0.867), and adding QTc yielded no incremental improvement (*Δ*AUC = 0.000, 95% CI: −0.012–0.024, *P* = 0.772; category-free NRI = 0.014; IDI = 0.0001). In the quartile analysis, MACE rates across Q1–Q4 were 29.0%, 22.6%, 38.7%, and 40.0%, respectively, with no significant trend (Cochran-Armitage *P* = 0.454). The diabetes subgroup association was attenuated (OR = 1.39, *P* = 0.744). The combined LVEF <40% and QTc >440 ms group had a cardiovascular MACE rate of 54.5%, compared with 21.2% in patients with neither risk factor (*P* = 0.016). These results are directionally consistent with the primary analysis and confirm that the incremental predictive value of QTc is limited when the endpoint is restricted to cardiovascular events.

#### Sensitivity analysis using the Fridericia formula

When all analyses were repeated using QTcF (QTcF = QT/RRⁱ⁄^3^), the mean QTcF was 426.4 ± 35.4 ms compared with 438.8 ± 38.0 ms by Bazett (mean difference 12.4 ms), and 32 patients (26.0%) exceeded 440 ms versus 47 (38.2%) by Bazett. The univariate association between QTc prolongation (>440 ms) and MACE was completely attenuated (OR = 1.23, 95% CI: 0.55–2.77, *P* = 0.615; continuous QTcF per 10 ms: OR = 1.04, 95% CI: 0.94–1.15, *P* = 0.428), in contrast to the significant Bazett-based finding (OR = 3.01, *P* = 0.004). In the multivariate model, reduced LVEF (OR = 0.75 per 5%, 95% CI: 0.60–0.93, *P* = 0.009) and elevated heart rate (OR = 1.64 per 10 bpm, 95% CI: 1.14–2.36, *P* = 0.008) remained independent predictors, while QTcF showed no association (OR = 1.01 per 10 ms, 95% CI: 0.90–1.13, *P* = 0.878). The full model AUC was 0.761 (95% CI: 0.672–0.849), with no improvement over the base model (*Δ*AUC = −0.001, DeLong *P* = 0.787; IDI = −0.0007, *P* = 0.735; optimism-corrected AUC = 0.740). The quartile pattern was non-monotone with no significant trend (Q1–Q4: 29.0%, 54.8%, 43.3%, 45.2%; Cochran-Armitage *P* = 0.354), and the previously significant diabetes subgroup association was abolished (OR = 1.71, *P* = 0.459). In the cardiovascular MACE sensitivity analysis using QTcF, LVEF (OR = 0.72, *P* = 0.005) and heart rate (OR = 1.46, *P* = 0.035) remained predictive while QTcF did not (OR = 1.00, *P* = 0.954), with *Δ*AUC = 0.000 (*P* = 0.926). These findings indicate that the univariate QTc–MACE association observed with the Bazett formula was substantially attributable to formula-induced overcorrection at high heart rates rather than true repolarization abnormalities.

Sensitivity analysis for missing data and BNP confounding. Among the 70 patients with available BNP data, the BNP–QTc Spearman correlation was weak and nonsignificant (r = 0.116, *P* = 0.340). In the complete-case seven-variable model incorporating BNP (standardized per SD), the QTc coefficient changed minimally compared with the six-variable model fitted in the same subset (OR per 10 ms: 1.14, 95% CI: 0.95–1.38, *P* = 0.168 vs. 1.13, 95% CI: 0.94–1.36, *P* = 0.195; coefficient change = 0.9%), and BNP itself was not independently significant (OR = 1.57 per SD, 95% CI: 0.86–2.89, *P* = 0.142; likelihood-ratio test *P* = 0.130; *Δ*AUC = 0.011). The BNP   ×   QTc interaction term was nonsignificant (OR = 0.94, *P* = 0.933). Multiple imputation (m = 20; imputation model including age, heart rate, LVEF, QTc, MACE, sex, and diabetes; R^2^ = 0.21) yielded pooled estimates consistent with the complete-case analysis (QTc: OR = 1.04 per 10 ms, 95% CI: 0.92–1.17, *P* = 0.577; BNP: OR = 1.56 per SD, 95% CI: 0.83–2.93, *P* = 0.171). Across four analytic scenarios (original six-variable model n = 123; six-variable model in BNP subset *n* = 70; seven-variable complete-case *n* = 70; and multiple imputation *n* = 123), QTc remained consistently nonsignificant (OR per 10 ms: 1.03–1.14, all *P* > 0.16), and the QTc coefficient changed by <1% after BNP adjustment. Creatinine and HbA1c, despite high missingness (63.4% and 77.2%), were not significantly associated with MACE in univariate analysis, making substantial residual confounding from these variables less likely. These findings indicate that BNP missingness does not materially confound the QTc–MACE association. Of note, BNP missingness was non-random: patients with missing BNP had lower MACE rates (26.4% vs. 55.7%, *P* = 0.002) and better LVEF (median 55% vs. 47%, *P* = 0.001), suggesting that BNP was preferentially measured in sicker patients; however, these concordant results provide reassurance but cannot fully exclude residual confounding given the potentially MNAR mechanism of BNP missingness.

### 3.5 QTc quartile groups and MACE risk

To further explore the association between QTc interval and MACE risk, exploratory quartile analysis was performed (Table 3, Figure 7). Patients were divided into four groups based on pre-procedural QTc quartiles: Q1 (348–416 ms, *n* = 31), Q2 (419–437 ms, *n* = 31), Q3 (438–460 ms, *n* = 31), and Q4 (462–619 ms, *n* = 30).

**Table 3:** QTc quartile groups and MACE risk.

Quartile	QTc Range (ms)	N	MACE, n (%)	Age, mean	LVEF, mean	Unadjusted OR (95% CI)	P
Q1	348–416	31	10 (32.3)	54.4	52.0	1.00 (Reference)	-
Q2	419–437	31	10 (32.3)	53.1	52.0	1.00 (0.34–2.90)	1.000
Q3	438–460	31	18 (58.1)	54.9	48.7	2.91 (1.03–8.20)	0.044
Q4	462–619	30	15 (50.0)	58.8	47.2	2.10 (0.74–5.93)	0.162

*P* for trend = 0.049 (Cochran-Armitage trend test); linear regression trend test *P* = 0.072; overall chi-square test *P* = 0.094. OR, odds ratio; CI, confidence interval. Q1, lowest QTc quartile (reference group).

**Figure 7:**
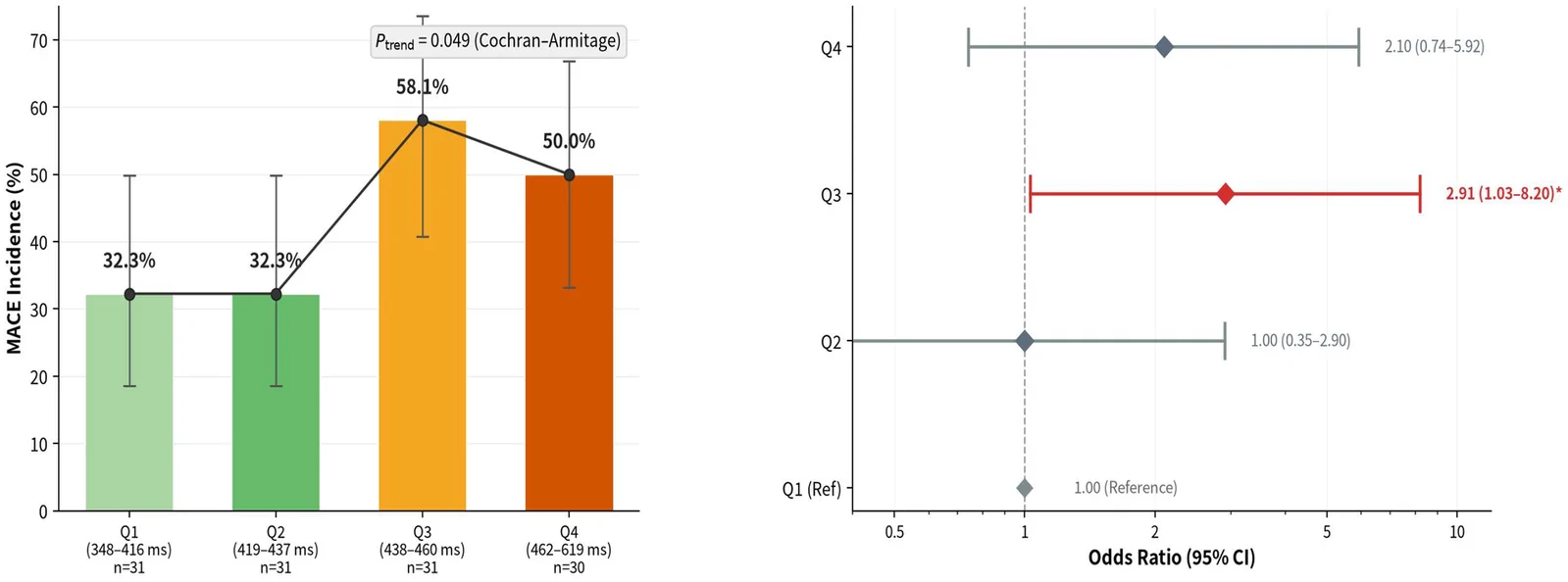
Exploratory quartile analysis of QTc and MACE risk. Line plot showing the incidence of in-hospital MACE across QTc quartiles with 95% confidence intervals. Quartile definitions: Q1, 348–416 ms (*n* = 31); Q2, 419–437 ms (*n* = 31); Q3, 438–460 ms (*n* = 31); Q4, 462–619 ms (*n* = 30). The P for trend was calculated using Cochran-Armitage trend test (*P* = 0.049). A more conservative test of linear association (point-biserial correlation between raw QTc values and MACE status) yielded r = 0.163 (*P* = 0.072), further supporting the absence of a linear dose-response relationship. MACE incidence across Q1–Q4 was 32.3%, 32.3%, 58.1%, and 50.0%, respectively. Q3 showed the highest unadjusted OR (2.91, *P* = 0.044). Note the non-monotonic pattern (Q4 < Q3); the Q3–Q4 difference was nonsignificant (*P* = 0.611), and this pattern should not be interpreted as evidence of a dose-response relationship.

MACE incidence across Q1 to Q4 was 32.3% (10/31), 32.3% (10/31), 58.1% (18/31), and 50.0% (15/30), respectively (Table 3, Figure 7). The Cochran-Armitage trend test yielded *P* = 0.049, but the linear regression trend test yielded *P* = 0.072 and the overall chi-square test *P* = 0.094; because the latter two did not reach significance and the pattern was non-monotonic, no reliable linear trend can be inferred. Using Q1 as the reference group, Q3 exhibited the highest unadjusted OR (OR = 2.91, 95% CI: 1.03–8.20, *P* = 0.044), while Q4 had an OR of 2.10 (95% CI: 0.74–5.93, *P* = 0.162). The MACE rate in Q4 (50.0%) was numerically lower than in Q3 (58.1%), and this difference was not statistically significant (Fisher exact *P* = 0.611). Moreover, the unadjusted OR for Q3 (*P* = 0.044) did not survive Bonferroni correction for three pairwise comparisons (adjusted *α* = 0.017). With only approximately 30 patients per group, statistical power is limited; the observed non-monotonic pattern likely reflects random variation and should not be interpreted as evidence of a dose-response relationship.

### 3.6 Relationship between LVEF and QTc interval

Given the significant association between LVEF and MACE, the relationship between LVEF and QTc interval was further explored. Scatter plot analysis revealed a weak inverse correlation between LVEF and QTc interval (r = −0.25, *P* = 0.005; Figure 8). Patients with both reduced LVEF (<40%) and prolonged QTc (>440 ms) had a numerically high MACE rate (9/11, 81.8%; 95% CI: 48.2%–97.7%),followed by those with a single risk factor only (LVEF <40% only: 60.0%; QTc >440 ms only: 52.8%), while those with neither risk factor had the lowest risk (28.8%; *P* = 0.002; Figure S2B). However, the LVEF–QTc interaction term was not statistically significant (OR = 1.09, 95% CI: 0.13–9.39, *P* = 0.941), indicating no evidence of multiplicative interaction beyond the additive effects of the two factors.

**Figure 8:**
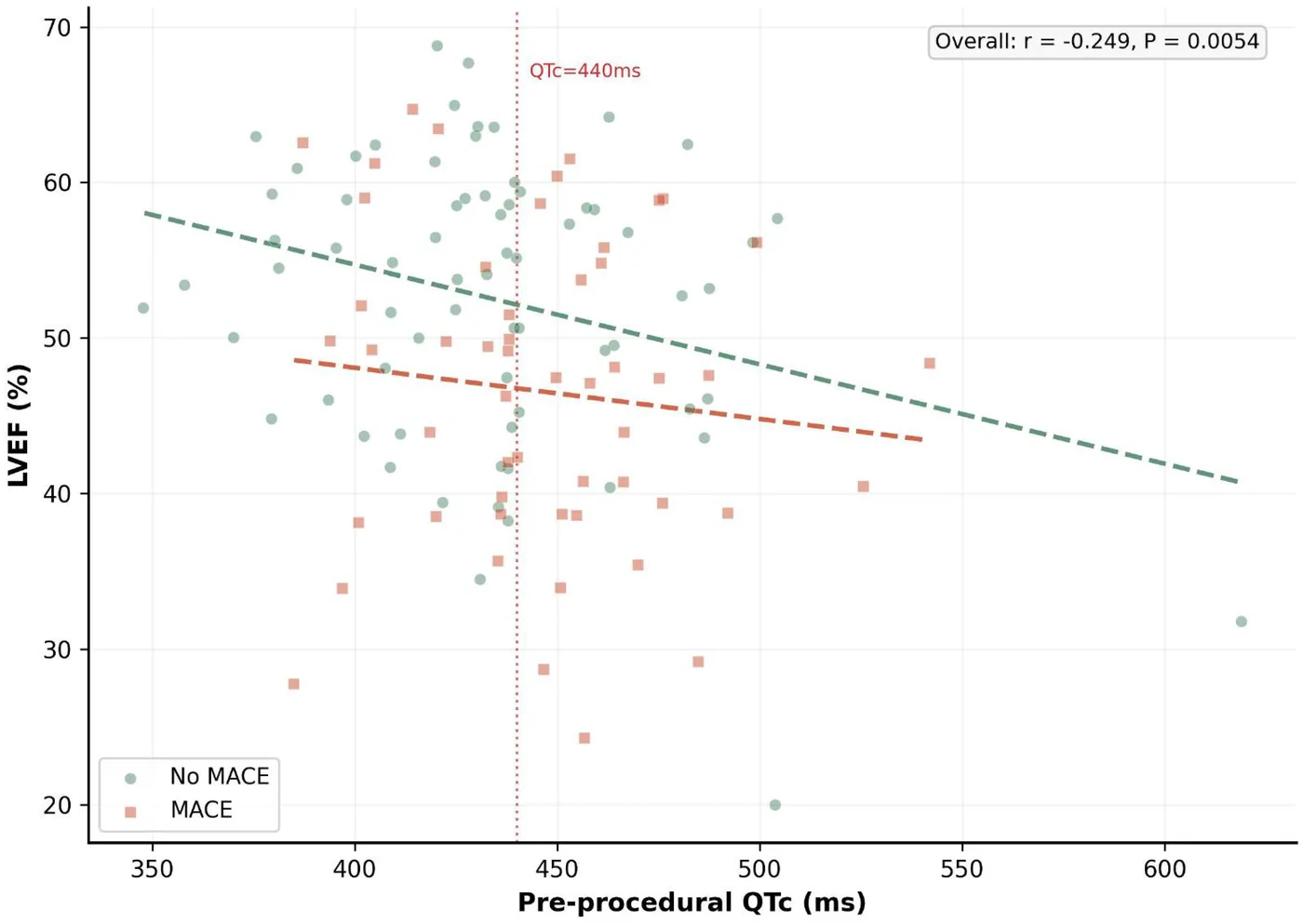
Relationship between left ventricular ejection fraction and QTc intervals. Scatter plot showing the correlation between left ventricular ejection fraction (LVEF) and pre-PCI QTc intervals. Patients who experienced in-hospital MACE are shown in red dots, and those without MACE are shown in blue dots. The linear regression line with 95% confidence interval band is displayed. A weak but statistically significant inverse correlation was observed (r = −0.25, *P* = 0.005).

### 3.7 Subgroup analysis

Subgroup analyses stratified by key clinical characteristics demonstrated that the association between prolonged QTc (>440 ms) and MACE risk was consistent across most subgroups (Figure 9). In the diabetes subgroup, patients with prolonged QTc had a significantly higher MACE rate compared with those with normal QTc (72.7% vs. 37.5%; OR = 4.44, 95% CI: 1.12–17.67, *P* = 0.047); in the non-diabetic subgroup, patients with prolonged QTc also showed a higher MACE rate compared with those with normal QTc (48.0% vs. 31.7%; OR = 1.99, 95% CI: 0.77–5.17, *P* = 0.216). In the age <65 years subgroup, prolonged QTc was significantly associated with MACE risk (58.8% vs. 32.3%; OR = 3.00, 95% CI: 1.26–7.13, *P* = 0.017); the association was similarly significant in the male subgroup (61.5% vs. 34.2%; OR = 3.07, 95% CI: 1.37–6.88, *P* = 0.009). A trend toward increased MACE risk with prolonged QTc was observed in the LVEF <50% subgroup (73.1% vs. 46.4%; OR = 3.13, 95% CI: 1.00–9.81, *P* = 0.057). All subgroup ORs exceeded 1, and no interaction reached statistical significance (all P for interaction >0.30).

**Figure 9:**
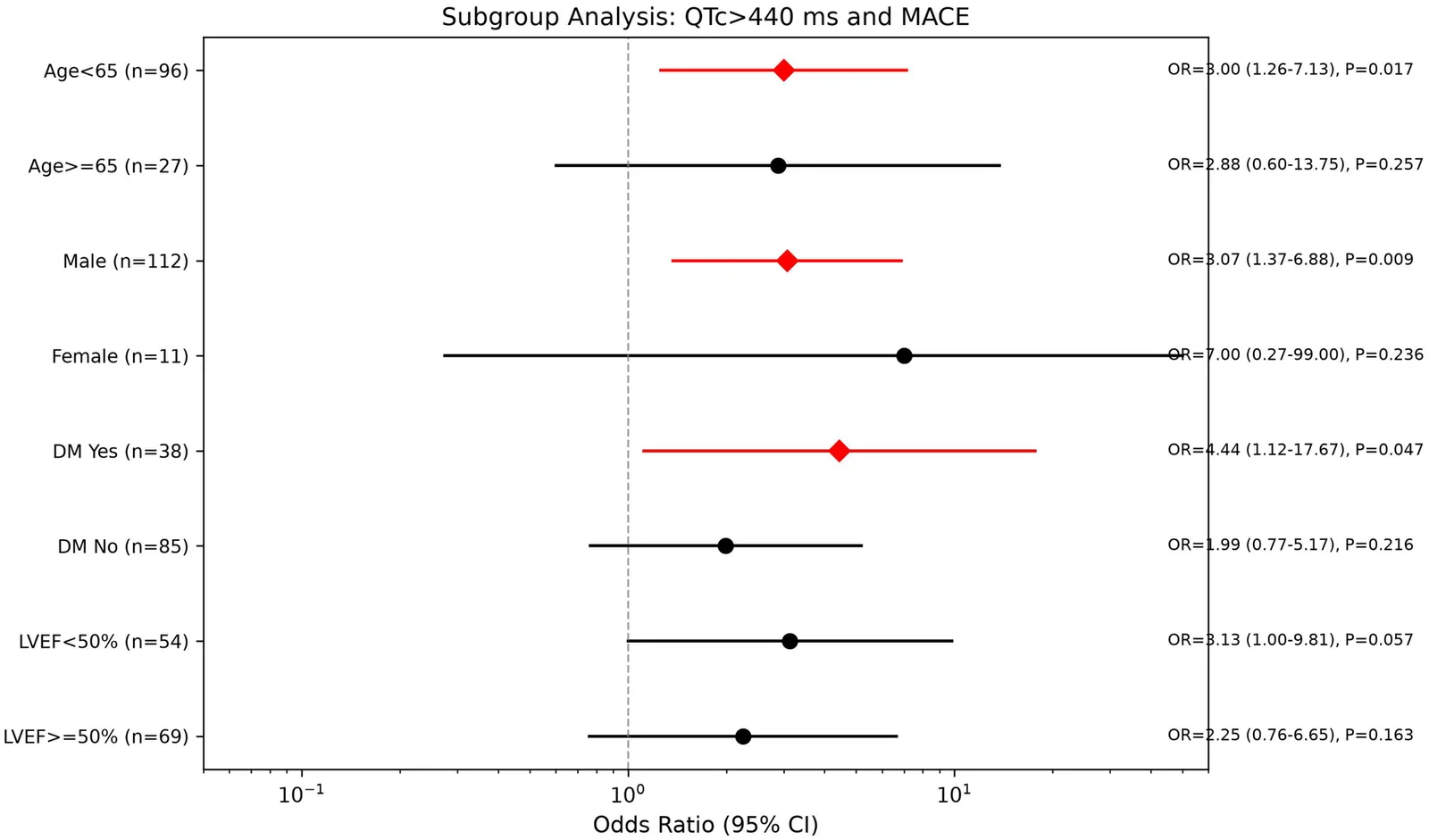
Subgroup analysis of the association between QTc prolongation (>440 ms) and in-hospital MACE. Forest plot displaying unadjusted odds ratios (OR) with 95% confidence intervals (CI) for the association between QTc prolongation and MACE risk across clinically relevant subgroups. All subgroup ORs were greater than 1, indicating a consistent direction of association. Statistically significant associations were observed in the age <65 years subgroup (OR = 3.00, *P* = 0.017), male subgroup (OR = 3.07, *P* = 0.009), and diabetes mellitus subgroup (OR = 4.44, *P* = 0.047). No significant interaction was found between any subgroup factor and QTc prolongation (all P for interaction >0.30).

### 3.8 Representative coronary angiographic findings

As a qualitative descriptive illustration of the range of pre-procedural coronary occlusion severity and post-procedural flow restoration observed in this cohort, four purposively selected angiographic cases are presented (Figure 10). These cases were not randomly sampled and are intended for descriptive illustration rather than quantitative inference. Case 1 (QTc 526 ms) exhibited complete LAD occlusion pre-PCI (TIMI 0 flow) and remained at TIMI 0 post-PCI (no-reflow), subsequently developing ventricular fibrillation. Case 2 (QTc 470 ms) had complete LAD occlusion pre-PCI with post-procedural flow restored to TIMI 3, but still experienced ventricular fibrillation. Case 3 (QTc 358 ms) presented with partial RCA stenosis pre-PCI (TIMI 1 flow) and achieved TIMI 3 flow post-PCI without MACE. Case 4 (QTc 619 ms) had complete LAD occlusion pre-PCI with post-procedural TIMI 3 restoration and no in-hospital MACE (Figure 10); this latter case serves as an explicit counterexample, indicating that extreme QTc prolongation does not invariably result in MACE and illustrating the heterogeneity of individual outcomes. These cases illustrate the culprit lesion site (indicated by red arrows) and the extent of post-PCI flow recovery. Collectively, these four cases qualitatively illustrate the broad spectrum of angiographic and clinical presentations in the cohort but do not by themselves establish a quantitative or causal association among pre-procedural QTc prolongation, TIMI flow grade, post-procedural no-reflow, and MACE occurrence.

**Figure 10:**
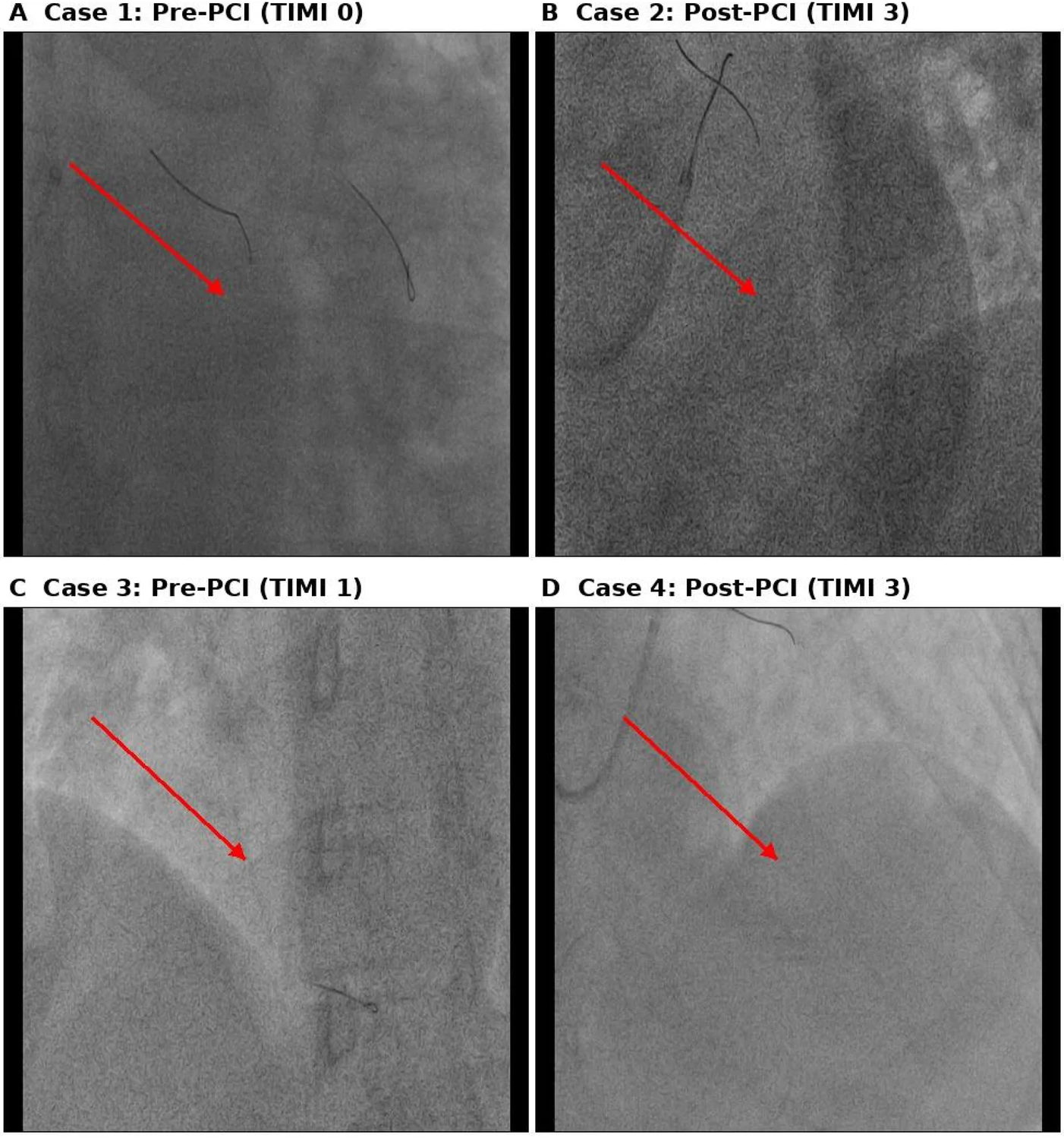
Representative coronary angiographic images from four typical cases. **(A)** Pre-PCI angiogram of a 38-year-old male patient (Case 1) with total LAD occlusion and TIMI 0 flow; this patient developed ventricular fibrillation post-PCI with a pre-procedural QTc of 526 ms. **(B)** Post-PCI angiogram of a 51-year-old male (Case 2) showing restored TIMI 3 flow after successful LAD recanalization; this patient experienced post-PCI ventricular fibrillation despite successful reperfusion, with a pre-procedural QTc of 470 ms. **(C)** Pre-PCI angiogram of a 54-year-old male (Case 3) with partial RCA occlusion and TIMI 1 flow; no MACE occurred during hospitalization (QTc 358 ms). **(D)** Post-PCI angiogram of a 76-year-old female (Case 4) demonstrating restored TIMI 3 flow after LAD intervention; no in-hospital MACE (QTc 619 ms). Red arrows indicate culprit lesion sites. Cases were purposively selected to span the observed range of TIMI flow grades, culprit vessels, and MACE outcomes; they are not a random or representative sample and are presented for descriptive illustration only.

## 4 Discussion

In this study of 123 patients with AMI undergoing primary PCI, we investigated the relationship between pre-procedural QTc interval and in-hospital MACE. Prolonged QTc (>440 ms) was significantly associated with MACE in univariate analysis (OR = 3.01, *P* = 0.004) but did not emerge as an independent predictor after adjustment for conventional risk factors (*P* = 0.605). Reduced LVEF (OR = 0.76, *P* = 0.019) and elevated baseline heart rate (OR = 1.57, *P* = 0.023) were identified as independent predictors. Exploratory quartile analysis showed numerically highest MACE risk in Q3 (58.1%, OR = 2.91, *P* = 0.044), but the three trend tests were inconsistent (Cochran-Armitage *P* = 0.049, linear regression *P* = 0.072, chi-square *P* = 0.094), the Q3–Q4 difference was nonsignificant (*P* = 0.611), and *P* = 0.044 did not survive Bonferroni correction; these findings should be regarded as hypothesis-generating only. LVEF showed a weak inverse correlation with QTc (r = −0.25). A numerically high MACE rate (81.8%) was observed in the 11 patients with both LVEF <40% and QTc >440 ms, but the interaction was nonsignificant (*P* = 0.941); this finding should be interpreted as exploratory. A significant risk-amplification effect of prolonged QTc was observed in the diabetes subgroup (OR = 4.44, *P* = 0.047) (Figure 2B), although the interaction did not reach statistical significance.

The in-hospital MACE rate in this study was 43.1%, which is higher than the approximately 19% reported in some comparable studies (11, 17). This difference is primarily attributable to the inclusion of hemorrhagic complications (BARC ≥ 2) in the MACE definition, with bleeding accounting for 24.5% of MACE events. To address the heterogeneity introduced by bleeding events, we performed a prespecified sensitivity analysis excluding 13 patients whose sole endpoint was hemorrhagic, yielding a cardiovascular MACE rate of 32.5% (40/123). In this sensitivity analysis, the univariate association between QTc prolongation and MACE was attenuated (OR = 2.07, *P* = 0.064 vs. OR = 3.01, *P* = 0.004 in the primary analysis), and the quartile trend (*P* = 0.454) and diabetes subgroup effect (*P* = 0.744) were no longer significant, suggesting that a portion of the apparent QTc signal in the primary analysis was contributed by bleeding events for which no direct electrophysiological pathway exists. In contrast, reduced LVEF remained a robust independent predictor (OR = 0.73, *P* = 0.008), and *Δ*AUC remained 0.000, confirming that the incremental value of QTc is negligible regardless of endpoint definition. These findings underscore the importance of using endpoint definitions that are biologically aligned with the exposure variable under investigation. Most comparable studies have restricted the primary outcome to “hard endpoints” such as death, reinfarction, and stroke; future studies with larger samples should adopt cardiovascular-specific composite endpoints and consider including bleeding as a separate safety outcome rather than within the primary efficacy composite.

The value of QTc prolongation as a predictor of cardiovascular events has been corroborated by multiple studies. A meta-analysis by Zhang et al. demonstrated that each 50-ms prolongation in QT interval was associated with a 20% increase in all-cause mortality (RR = 1.20). In AMI patients, Lin et al. found that QTc >445 ms independently predicted all-cause death and heart failure readmission within 2 years, and Mann et al., analyzing 1,054 STEMI patients, confirmed that admission QT prolongation was associated with increased mortality. The univariate OR of 3.01 for prolonged QTc observed in this study is directionally consistent with these reports, but the failure to achieve independent significance after multivariate adjustment may be attributable to the limited sample size, the use of in-hospital MACE rather than long-term mortality as the endpoint, and collinearity among QTc, LVEF, and heart rate. The AUC of 0.767 in our composite model is comparable to the QTcSar score AUC of 0.734 reported by Li et al. and the model AUC of 0.74 reported by de Bruyne et al. (7, 19).

Recent studies have further supported the superiority of a multi-parameter combined approach. Gandamsetty et al. demonstrated that a prolonged baseline Tp-e interval (≥98 ms) independently predicted early arrhythmic events within 48 h in patients with acute MI (adjusted OR = 1.024, AUC = 0.736), and the Tp-e/QT ratio provided additional prognostic discrimination, with nonsurvivors exhibiting significantly higher values alongside reduced LVEF. Li et al. (2023) similarly found that combining QTc with clinical parameters enhanced predictive accuracy, which is directionally consistent with our findings. Evidence regarding the dynamic changes of QTc/QTd during reperfusion, the influence of electrolyte disturbances on QTc, and the independent prognostic value of QTc in lead aVR also provides multidimensional support for the application of QTc in the AMI setting. More recently, Dal and Eraybar reported in 567 STEMI patients undergoing primary PCI that the Tp-e/QTc ratio (cutoff 0.190) predicted 90-day MACE with an AUC of 0.743, supporting the prognostic utility of combined repolarization indices in the acute PCI setting (4); similarly, Li et al. developed and internally validated a LASSO-derived nomogram incorporating LVEF, Sema4D, QTd, QRS duration and QTc in 321 STEMI patients (AUC = 0.87), demonstrating that QTc contributes meaningfully only within broader multi-marker models (33). These 2025 reports are directionally aligned with the present finding that the isolated incremental value of a single QTc measurement beyond conventional clinical predictors is negligible in this single-center cohort (*Δ*AUC = 0.001, DeLong *P* = 0.888), supporting the repositioning of this study as a validation of the limited incremental value of QTc within multivariable models rather than as a standalone predictor (34–37).

In the quartile analysis of this study, Q3 exhibited the highest MACE risk (58.1%, OR = 2.91, *P* = 0.044), yet the MACE rate in Q4 (50.0%) was lower than in Q3, presenting a non-monotonic pattern. It should be noted that the trend test results are method-dependent (the three methods yielding partially inconsistent conclusions). Furthermore, the Q3 unadjusted *P* = 0.044 did not survive Bonferroni correction for three pairwise comparisons (adjusted *α* = 0.017), and the direct Q3-vs-Q4 comparison was nonsignificant (Fisher exact *P* = 0.611). Potential explanations for the non-monotonic pattern include: (1) limited sample size in Q4 (*n* = 30), resulting in insufficient statistical power and wide confidence intervals (95% CI: 0.74–5.93); (2) patients with extremely prolonged QTc may have received more aggressive prophylactic interventions before PCI, thereby preventing some MACE events; and (3) the heterogeneity of the MACE composite endpoint may lead to different event-type compositions across QTc intervals. The difference between Q3 and Q4 was not statistically significant, and the non-monotonic pattern may simply reflect random variation. Therefore, the quartile analysis in this study should be regarded as exploratory and should not be interpreted as evidence of a dose-response relationship. Robbins et al., in the Cardiovascular Health Study (CHS), identified a continuous dose-response relationship between QT interval and mortality; however, that study had a substantially larger sample and a long-term mortality endpoint. Future studies with larger samples should employ restricted cubic spline (RCS) analysis to precisely characterize the QTc-MACE relationship (5).

Reduced LVEF was one of the strongest independent predictors (OR = 0.76 per 5% increment, *P* = 0.019), consistent with the findings of Zabel et al. and Huang et al. LVEF was weakly inversely correlated with QTc (r = −0.25), and MACE risk was highest (81.8%) when both parameters were abnormal, numerically exceeding the risk in patients with a single abnormality and those with neither (28.8%). However, the LVEF–QTc interaction term was nonsignificant (OR = 1.09, *P* = 0.941), and the dual-abnormality subgroup comprised only 11 patients (95% CI for 81.8%: 48.2%–97.7%); therefore, this observation should not be interpreted as evidence of a synergistic or supra-additive effect. However, incremental AUC analysis showed that adding QTc yielded a *Δ*AUC of only 0.001 (*P* = 0.888), with nonsignificant NRI and IDI, indicating that the incremental discriminatory value of QTc is limited, and the combined risk stratification hypothesis requires validation in larger prospective studies. Elevated baseline heart rate was also an independent predictor (OR = 1.57 per 10 bpm, *P* = 0.023), consistent with the pathophysiological mechanism of sympathetic activation in the acute phase of AMI (18, 38).

The risk-amplification effect of prolonged QTc in the diabetes subgroup reached statistical significance (OR = 4.44, *P* = 0.047), whereas the OR in the non-diabetic subgroup was 1.99 (*P* = 0.216); however, the interaction was not statistically significant (*P* = 0.349). In the multivariate model, diabetes also did not achieve independent significance (OR = 2.12, *P* = 0.103), which may reflect the limited sample size. Mechanistically, diabetes affects ventricular repolarization through multiple pathways, including downregulation of PI3 K signaling and cardiac autonomic neuropathy. Cox et al., Cardoso et al., and Okin et al. independently confirmed that QTc parameters have long-term prognostic value in patients with type 2 diabetes. The nonsignificant interaction suggests that the combined effect is not a simple multiplicative interaction, and larger studies are needed to clarify this relationship (39–44).

The four representative cases in this study (Figure 10) qualitatively illustrate the broad spectrum of coronary disease severity and QTc values: shorter QTc and higher TIMI grades were associated with more favorable outcomes. Because these cases were purposively selected to span the range of observed outcomes rather than randomly sampled, no quantitative inference regarding the QTc–TIMI–MACE relationship should be drawn from them; all quantitative findings are based on the full cohort analyses reported above. Previous studies have also demonstrated that the no-reflow phenomenon is closely related to QT dispersion, and QTc prolongation may partly increase MACE risk by reflecting microvascular dysfunction and myocardial reperfusion injury. From a pathophysiological standpoint, QTc prolongation reflects increased ventricular repolarization dispersion, providing the electrophysiological substrate for reentrant ventricular arrhythmias. Smoczyńska et al., in the EU-CERT-ICD study, demonstrated that short-term QT interval variability significantly increased before ventricular arrhythmia onset. In the present study, acute heart failure and ventricular arrhythmias were the predominant MACE types (accounting for 37.7% and 35.8%, respectively), supporting this mechanism (45–48).

The loss of independent significance for QTc in the multivariate model warrants discussion. First, LVEF and QTc share pathophysiological links — myocardial injury leads to repolarization prolongation, and the inclusion of LVEF in the model may partially absorb the predictive effect of QTc, resulting in overlapping information content. Second, the Bazett formula systematically overestimates QTc at high heart rates (25, 26); our Fridericia sensitivity analysis confirmed and quantified this bias. Among 47 patients classified as QTc-prolonged by Bazett, 16 (34.0%) were reclassified as normal by Fridericia; these patients had markedly higher heart rates (87.3 vs. 73.1 bpm), and Bazett overestimated QTc by up to 36 ms at HR ≥ 90 bpm. Critically, 14 of these 16 reclassified patients (87.5%) experienced MACE, indicating that the high event rate in the Bazett-prolonged group was driven in part by tachycardia itself—an independent MACE predictor—rather than by true repolarization delay. Correspondingly, the significant univariate QTc–MACE association observed with Bazett (OR = 3.01, *P* = 0.004) was completely abolished using Fridericia (OR = 1.23, *P* = 0.615), and the diabetes subgroup signal (*P* = 0.047 with Bazett) was likewise eliminated (*P* = 0.459). QTcB retained a significant positive correlation with heart rate (r = 0.287, *P* = 0.001), whereas QTcF did not (r = −0.021, *P* = 0.821). These results strongly suggest that a substantial component of the apparent univariate QTc signal was an artifact of Bazett overcorrection in tachycardic patients, who are also at higher MACE risk due to sympathetic activation—an interpretation consistent with the Guangzhou Biobank cohort finding that Bazett was the poorest-performing correction formula and that Bazett-corrected QTc was not associated with incident cardiovascular disease (42). In contrast, LVEF and heart rate remained robust independent predictors regardless of the correction formula, reinforcing that the multivariate null finding for QTc is not formula-dependent. Third, QTc and LVEF reflect distinct dimensions — ventricular electrophysiology and mechanical function, respectively — and their interaction may be more complex than simple linear addition (4).

### Study limitations

This study has several limitations. (1) The single-center retrospective design may introduce selection and information bias, limiting generalizability. (2) No formal *a priori* sample size calculation was performed. The sample size (*n* = 123) was limited, with an EPV of 8.8, below the recommended threshold; therefore, the multivariate findings should be regarded as exploratory and hypothesis-generating rather than confirmatory, requiring validation in adequately powered prospective cohorts. (3) Although the six primary model variables had no missing values (*n* = 123 for the primary multivariate analysis), secondary laboratory parameters had high missingness (BNP: 43.1%, creatinine: 63.4%, HbA1c: 77.2%). Sensitivity analyses using complete-case seven-variable modeling and multiple imputation (m = 20) showed that BNP adjustment did not materially alter the QTc coefficient (change <1%), with no significant BNP–QTc interaction (*P* = 0.933); however, BNP missingness was non-random (MNAR; *P* = 0.002 for MACE difference), and the imputation model assumed MAR, so residual selection bias cannot be entirely excluded. The BNP–QTc interaction reported in stroke-heart syndrome (HR = 4.92) (33) was not replicated in this AMI cohort, possibly owing to population differences and limited power. (4) Although the Fridericia sensitivity analysis suggested that a portion of the univariate Bazett-based QTc signal was attributable to formula overcorrection in tachycardic patients (25, 26), the study was not designed to determine which correction formula is optimal in AMI; future prospective studies with simultaneous RR-interval recording and head-to-head comparison of multiple correction formulas (Bazett, Fridericia, Framingham, and Hodges) are warranted (5). Some LVEF data were derived from discharge summaries rather than a standardized echocardiographic protocol (6). STEMI and NSTEMI were not analyzed as separate subgroups (7). The study population included only 11 female patients (9.1%), resulting in extreme sex imbalance (8). The endpoint was in-hospital MACE without long-term follow-up (9). Potential confounders that could influence QTc duration or MACE risk were not systematically collected or adjusted for, including Killip class on admission (used solely for endpoint adjudication), use of QTc-prolonging medications (*β*-blockers, amiodarone), serum potassium and magnesium levels, and infarct location; similarly, smoking pack-years were not recorded, precluding dose-response assessment of tobacco exposure. These residual confounders may have biased the QTc–MACE association in either direction (10). The representative coronary angiographic cases were presented for descriptive illustration only, without systematic quantitative analysis (29, 30). Accordingly, the concordance between complete-case and imputation analyses should be interpreted as reassuring but not definitive, given that MAR-based imputation cannot fully correct for MNAR-driven missingness.

In conclusion, pre-procedural QTc prolongation is significantly associated with in-hospital MACE in AMI patients undergoing primary PCI but does not serve as an independent predictor after adjustment for conventional risk factors, with limited incremental discriminatory value (*Δ*AUC = 0.001, *P* = 0.888). Given the single-center retrospective design, limited sample size, and absence of a formal *a priori* power calculation, these findings should be regarded as exploratory and hypothesis-generating. Reduced LVEF and elevated baseline heart rate are independent predictors of MACE, and a numerically high MACE rate was observed when both LVEF reduction and QTc prolongation coexisted (9/11, 81.8%), although the interaction was nonsignificant (*P* = 0.941) and this exploratory finding requires validation in larger cohorts. Future multicenter prospective studies should: (1) validate the predictive value of QTc in adequately powered samples using heart-rate-independent correction formulas such as Fridericia's, as the present sensitivity analysis suggests that Bazett-based univariate QTc–MACE associations should be interpreted with caution in tachycardic AMI populations; (2) compare multiple correction formulas (Bazett, Fridericia, Framingham, and Hodges) in AMI-specific populations; (3) use restricted cubic spline analysis to precisely characterize the QTc-MACE dose-response relationship; and (4) explore comprehensive multi-marker predictive models that combine QTc with novel repolarization indices such as the Tp-e/QTc ratio (36) and broader biomarker panels (e.g., Sema4D, QTd, QRS duration), given recent evidence that combined repolarization and multi-marker nomograms outperform QTc alone for post-PCI risk prediction (37).

## Data Availability

The raw data supporting the conclusions of this article will be made available by the authors, without undue reservation.
